# Structural insights into the potential binding sites of Cathepsin D using molecular modelling techniques

**DOI:** 10.1007/s00726-023-03367-1

**Published:** 2024-04-22

**Authors:** Subodh A. Kamble, Sagar S. Barale, Ali Abdulmawjood Mohammed, Sneha B. Paymal, Nitin M. Naik, Kailas D. Sonawane

**Affiliations:** 1 Structural Bioinformatics Unit, Department of Biochemistry, Shivaji University, Kolhapur, M.S. 416004 India; 2 Department of Microbiology, Shivaji University, 416004 M.S. Kolhapur, India; 3 Department of Chemistry, Shivaji University, Kolhapur, M.S. 416004 India

**Keywords:** Alzheimer’s disease (AD), Amyloid beta (Aβ), Cathepsin D (CathD), Virtual screening, MD simulation, MM-GBSA

## Abstract

Alzheimer’s disease (AD) is the most prevalent type of dementia caused by the accumulation of amyloid beta (Aβ) peptides. The extracellular deposition of Aβ peptides in human AD brain causes neuronal death. Therefore, it has been found that Aβ peptide degradation is a possible therapeutic target for AD. CathD has been known to breakdown amyloid beta peptides. However, the structural role of CathD is not yet clear. Hence, for the purpose of gaining a deeper comprehension of the structure of CathD, the present computational investigation was performed using virtual screening technique to predict CathD's active site residues and substrate binding mode. Ligand-based virtual screening was implemented on small molecules from ZINC database against crystal structure of CathD. Further, molecular docking was utilised to investigate the binding mechanism of CathD with substrates and virtually screened inhibitors. Localised compounds obtained through screening performed by PyRx and AutoDock 4.2 with CathD receptor and the compounds having highest binding affinities were picked as; ZINC00601317, ZINC04214975 and ZINCC12500925 as our top choices. The hydrophobic residues Viz*.* Gly35, Val31, Thr34, Gly128, Ile124 and Ala13 help stabilising the CathD-ligand complexes, which in turn emphasises substrate and inhibitor selectivity. Further, MM-GBSA approach has been used to calculate binding free energy between CathD and selected compounds. Therefore, it would be beneficial to understand the active site pocket of CathD with the assistance of these discoveries. Thus, the present study would be helpful to identify active site pocket of CathD, which could be beneficial to develop novel therapeutic strategies for the AD.

## Introduction

The Greek word cathepsin means “to digest” as reported earlier (Wilstätter and Bamann 1928). Cathepsin D (CathD) key activities depend on the restricted proteolysis of physiologically active protein rather than bulk breakdown in lysosomes (Saftig et al. 1995). Endosomal/lysosomal disruption in neurons activates microglia, which triggers an inflammatory response and neurodegeneration (Soetikno et al. 2003). Activated microglia produce cathepsins that degrade extracellular matrix proteins, killing neurons as discussed previously. It has been reported that Cathepsin D clears peptides from human and rat brains (Hamazaki 1996; McDermott and Gibson 1997). Microglia cells have been shown enhanced uptake of Aβ peptides via class A and B scavenger receptor type I (Paresce et al. 1996; Husemann et al. 2001). Further, microglia lysosomes collect and breakdown Aβ peptides (Paresce et al. 1997). It has been found that pepstatin A inhibits the breakdown of microglia Aβ peptide (Kakimura et al. 2002). These data show that CathD degrades phagocytosed peptides in microglial lysosomes. It has been proved that immunising transgenic mice with these peptides help reduce plaques (Schenk et al. 1999). Thus, phagocytosis and subsequent peptide breakdown by microglia may play a key role in Alzheimer’s immunotherapy. Similarly, the role of various enzymes in the degradation of amyloid beta peptides has also been discussed in earlier studies (Barale et al. 2019; Dhanavade and Sonawane 2020).

Since the 1990s, new docking techniques have allowed scientists to anticipate ligand binding mode and precise intermolecular interactions in and around the active site (Kitchen et al. 2004). Virtual screening allows a library of small compounds to be screened against a target quickly and precisely (Ou-yang et al. 2012; Jalkute et al. 2015a, b). Docking and virtual screening techniques have been automated and enhanced to predict ligand binding mechanism and enzyme catalytic location (de Graaf et al. 2005). Babu and co-workers in 2012 have worked on CathD inhibitors (Stamford et al. 2012).

Here, in the present manuscript, we used a ligand-based drug discovery approach to find the lead compounds that had physicochemical properties with well-known CathD ligands. Using Dockblaster, ZINC's small molecule library was examined as per earlier reports (Irwin and Shoichet 2005; Irwin et al. 2009). Localised compounds from CathD screening by AutoDock 4.2, from which we selected ZINC00601317, ZINC04214975, ZINCC12500925 and ZINC04215004 screened compounds having highest binding affinities. The screened four compounds’ IUPAC nomenclature is as follows: ZINC00601317 (1-(3-cyano-3,3-diphenylpropyl)-4-phenylpiperidine-4-carboxylic acid); ZINC04214975 (Ethyl 1-[2-(benzyloxy)ethyl]- 4-phenylpiperidine- 4-carboxylate); ZINCC12500925 (1C3-4-[-4-(2-oxo-3-propanoylbenzimidazol-1-yl)piperidin-2-tun-1-yl)-2,2-diphenylbutanenitrile; ZINCC04215004 (6C42-[-1,3-dimethyl-2,6-die-4,5,4,5-tetrahydropurin-2-yl)ethyl-[-1-phenylpropan-2-yl]azantu. Hydrophobic residues Gly35, Val31, Thr34, Gly128, Ile124 and Ala13 emphasise substrate and inhibitor specificity by stabilising CathD-ligand complexes. Thus, these findings would be helpful to understand the active site pocket and substrate binding of CathD.

## Methodology

### Structural refinement of CathD and virtual screening

The high-resolution crystal structure of human CathD was retrieved from RCSB (Research Collaboratory for Structural Bioinformatics) structural database having Protein data Bank (PDB) ID 1LYA (Baldwin et al. 1993). The heteroatoms and water molecules from the retrieved structure were removed and prepared for virtual screening by adding polar hydrogen and necessary charges generated in Chimera1.15. The missing side chains of residue Gln97 were built using chimera. Dockblaster tool was used to perform virtual screening against 35 million compounds present in the ZINC library (Irwin et al. 2009; Shoichet 2004). The flexible ligand sampling algorithm in Dock Blaster superimposes atoms of the docked molecule as per the given binding site (Irwin et al. 2009). The ligand selection was performed by bin size; distance tolerance up to 2.0 Å. Also, each ligand configuration was scored for electrostatics, van der Waals interaction complementarity and corrected for ligand desolvation. The selected hits were transferred through Lipinski filter using FAF2/ADME/tox server (Lagorce et al. 2008). The ligands which satisfy Lipinski rules were selected for further docking process. All 100 molecules were obeying Lipinski rule that is RO5 (MWT ≤ 500, log *P* ≤ 5, H-bond donors ≤ 5, and H-bond acceptor ≤ 10) that depicted lead like potency (Lipinski et al 2001). Furthermore, certain ligands are not included because of their high internal energy, conformational flexibility, novelty, and diversity (Lipinski et al. 2001; Carlsson et al. 2011). The high scoring ligand conformation is minimised with 100 steps of simple rigid body minimization (Irwin et al. 2009). In the next phase, we selected top 100 hits based on binding energy for more precise docking using optimised parameters by generating maximum number of conformations per ligands and increased flexibility.

### Molecular docking using AutoDock

The PyRx virtual screening software was used for local rigid docking of the top screened 100 compounds from the Dock blaster binding energy. The purpose of local docking after the blind docking in *Dock blaster* is to eliminate unwanted hits and to get best possible compound targeting the specified binding cavity. The compounds that follow the Lipinski’s rule (Lipinski et al. 2001; Lagorce et al. 2008; Carlsson et al. 2011) were considered for the local docking. Further, top four hits from the PyRx local docking were chosen for molecular docking study using AutoDock 4.2 to gain detailed insights to their intermolecular interactions. The grid map was generated using ‘autogrid’ programme (Morris et al. 2009) and grid dimension was set to 60 × 60 × 60 points with grid spacing of 0.375 Å. The docking was run for 100 conformations per ligands with 27,000 generations using Lamarkian Genetic algorithm.

The best ranked conformations were identified by clustering all the poses with the rmsd cut-off value of 2.0 Å. The binding pocket residues namely Asp33, Asp126, Asp231, Ser36, Ser80, Thr234, Thr205, Gly233, Gly81 were kept flexible to get a reasonable and precise binding mode. The redocking was performed for ligands ZINC00601317, ZINC04214975, ZINCC12500925 and ZINCC04215004 having least binding energy and RMSD values. The most stable four screened lead molecules were further subjected for all atom MD simulation in explicit solvent. These four compounds ZINC00601317, ZINC04214975, ZINCC04215004 and ZINCC12500925 are hereafter referred as C1, C2, C3 and C4, respectively, for the simplicity.

### Molecular dynamic (MD) simulation study

MD simulation has been successfully used to investigate molecular interactions and binding mode of several enzyme-ligand complexes (Dhanavade et al. 2013; 2014; 2016; Sonawane and Barage 2015; Kamble et al. 2021; Jalkute et al. 2015b; Ali et al. 2023; Paymal et al. 2023). The complexes CathD-C1, CathD-C2, CathD-C3 and CathD-C4 were selected for MD simulations as they expressed stable non-bonded interactions. The MD simulations were done using GROMACS software package (Spoel et al. 2005). The partial charges of ligands were generated using antechamber module of ambertools18. The topologies of the complexes were built using xleap with the *‘ff99SBildn’* force field. The complexes were solvated using TIP3P water mode and required number of counter ions were added to neutralise the charges on the prepared systems. The topology of the whole solvated complexes was recorded in xleap module of ambertools18 and converted to gromacs compatible file format using ParmEd. The energy minimization was done using steepest descent method for all the complexes with maximum force tolerance of 1000 kJ/mol/nm. Particle Mesh Ewald (PME) constraint solver (LINCS) algorithm was used for bond constraints. All the systems were equilibrated using NVT and NPT ensembles for 1 ns each to maintain temperature and pressure constant at 300 K and 1 bar respectively. The cut-off radii for coulomb and van der Waals (VDW) interactions were set to 10 Å for all complexes. The final production MD run was continued for 100 ns for each protein–ligand complex and trajectory files were saved using GROMACS utilities for further analysis. The trajectories obtained were analysed using in-build GROMACS utilities for structural stability parameters such as RMSD, RMSF, Rg, SASA, etc. and intermolecular interactions.

### Binding free energy calculations

The binding affinity between CathD and compounds screened (C1 to C4) was estimated by calculating binding free energies using the recently released tool ‘*gms_MMGBSA’* (Valdés-Tresanco et al. 2021)*.* Further, per residue decomposition energy was also calculated to identify the key significant residues from the binding pocket those would favour the stable complex formation. A stable trajectory observed between the 0–100 ns of simulation has been used for this binding energy calculation, i.e. 50–100 ns using some of the representative frames with the interval of 60. In the present calculation, we ignored the entropic contribution as it is computationally very expensive. The approach MM-GBSA uses implicit solvation methods for calculation in which we have set dielectric constant 2 for considering the solvent effect. For present study, the entropy contributions are not taken into consideration for energy calculations.

The individual components of the binding energy has been calculated using following equations,

The free binding energy for a complex can be estimated as follows:

$$ \Delta G_{bind} \, = \,\langle G_{COM} \rangle \, - \,\langle G_{REC} \rangle \, - \,\langle G_{LIG} \rangle $$

In turn, ΔGbind can also be represented as:

$$ \Delta G_{bind} \, = \,\Delta H\, - \,T\Delta S $$

The ΔH can be decomposed into different terms:

$$ \Delta H\, = \,\Delta E_{MM} \, + \Delta G_{sol} $$

where:

$$ \Delta E_{MM} = \,\Delta E_{bonded} \, + \,\Delta E_{nonbonded} \, = \,(\Delta E_{bond} \, + \,\Delta E_{angle} \, + \,\Delta E_{\Delta edral} )\, + \,(\Delta E_{ele} \, + \Delta E_{vdW} ) $$

and

$$ \Delta G_{sol} \, = \,\Delta G_{polar} + \,\Delta G_{non - polar} \, = \,\Delta G_{{{{GB} \mathord{\left/ {\vphantom {{GB} {PB}}} \right. \kern-0pt} {PB}}}} \, + \,\Delta_{Gnon - polar} $$

## Results and discussion

### Virtual screening study

**Fig. 1:**
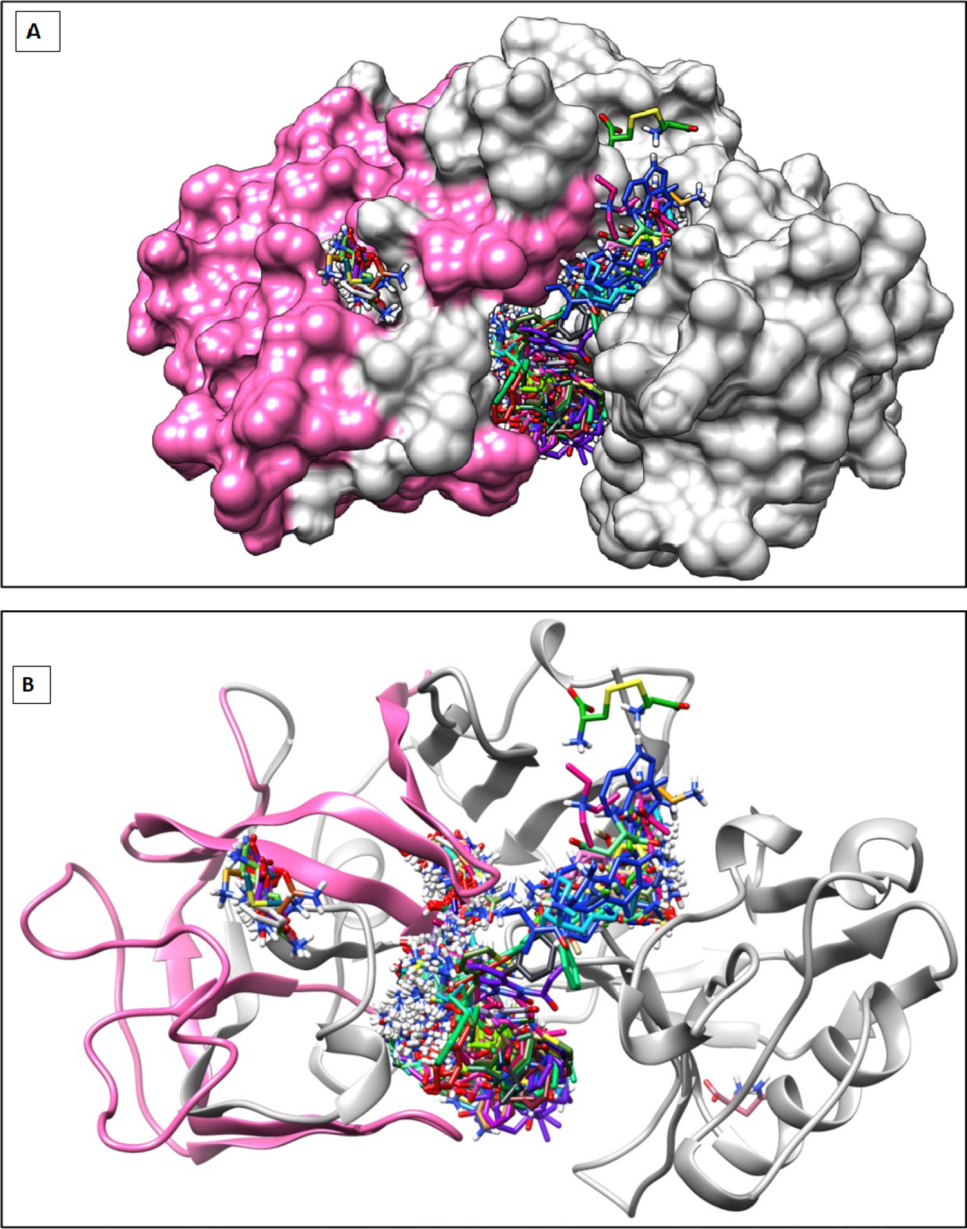
**A** Surface representation of docked complex of human CathD with 100 ligands by ZINC data base, docked deeply inside the cavity of CathD. **B** Chain representation of docked complex of human CathD chain A (hot pink) colour chain B (grey) colour with 100 ligands obtained using ZINC data base, docked deeply inside the cavity of CathD.

Virtual screening technique was used to identify the putative binding pockets of CathD and respective interacting residues. It has been reported that the interface between the heterodimeric subunits, i.e. chains A and B, forms deep and wide cavity could serve as the possible binding pocket of the CathD. In our virtual screening, most of the compounds favour the binding pocket region reported earlier at the interface of chains A and B. The larger compounds show extended conformations, whilst small sized molecules get deeply buried in the cavity. Tables 1 and 2 represents the top 100 compounds with their respective binding free energy values (ΔG) in descending order. The binding mode of top 100 compounds has been shown in Fig. 4A and B. The top few complexes were analysed in detail to gain more structural insights to the binding pocket residues of CathD. It has been observed that interface residues Gly35, Val31, Thr34, Gly128, Ile124, Ala13, Asp33,Tyr78, Asp231, Ser36, Ser80, Thr234, Thr205, Gly233, Gly81 form stable H-bonding interactions and could play crucial role in stabilising these complexes. Further, physicochemical properties of the top 100 compounds have been analysed using ADMETLab2.0. Interestingly, we observed that not only these 100 compounds favour the binding pocket but also showed the acceptable drug like (Lipinskies rule) and ADME properties. Tables 1, 2 and 3 represent the physicochemical, pharmacokinetic and drug likeliness properties of the selected top 100 molecules Fig. 1.

**Table 1:** Dock score of zinc ligands with whole CathD given by Dockblaster with their respective binding free energy values (ΔG)

Zinc database ID		
C00000092-38.17	C01587665-38.97	C05458899-42.51
C00000293-39.15	C01608965-40.22	C05650525-41.60
C00000294-38.33	C01664586-40.34	C05843973-38.23
C00000609-37.35	C01664587-42.32	C06482440-38.48
C00000859-37.64	C01841010-37.79	C08221225-33.47
C00001026-37.35	C01849735-37.93	C12484881-45.91
C00001612-37.98	C01850015-38.24	C12484909-39.08
C00001732-38.46	C01850017-40.11	C12495103-40.98
C00049153-34.88	C01850106-41.77	C12500917-42.62
C00083315-40.39	C01850108-39.01	C12500925-38.35
C00105196-35.68	C01850113-37.50	C12500934-43.56
C00139370-39.46	C01850151-40.88	C12500936-43.57
C00404450-38.34	C01850153-37.71	C13513942-35.19
C00404451-38.21	C01851048-37.44	C13513943-35.91
C00537752-43.31	C02007678-38.31	
C00601281-42.46	C02007680-40.60	
C00601317-37.80	C02007682-40.07	
C00608179-37.64	C02522669-39.12	
C00895032-33.50	C02583773-40.82	
C00895034-33.43	C03830195-38.08	
C00968306-38.11	C03830716-45.01	
C00968311-37.53	C03830891-36.56	
C01530763-39.83	C03831014-38.17	
C01530767-39.44	C03875336-39.88	
C01532129-47.08	C04213409-38.64	
C01532130-57.63	C04214975-41.34	
C01532131-48.83	C04214989-39.73	
C01532132-51.30	C04215004-38.87	
C01532522-43.70	C04215196-42.02	
C01532525-45.84	C04215955-38.54	
C01532526-36.79	C04215962-37.88	
C01532529-33.17	C04228231-45.74	
C01532530-40.74	C05458868-37.76	
C01532556-39.26	C05458875-40.26	
C01532614-39.14	C05458878-42.73	
C01532673-49.49	C05458886-39.86	
C01532734-35.55	C05458893-37.83

**Table 2:** Binding free energy values of individual compounds given by the PyRx docking

Zinc database ID NO	Energy Kcal\mol	Zinc database ID NO	Energy Kcal\mol
C00000092-38.17	– 5.5	C01850108-39.01	– 7.1
C00000293-39.15	– 6.5	C01850113-37.50	– 7.9
C00000294-38.33	– 6.1	C01850151-40.88	– 8.4
C00000609-37.35	– 5.5	C01850153-37.71	– 7.6
C00000859-37.64	– 6	C01851048-37.44	– 8.2
C00001026-37.35	– 6.3	C02007678-38.31	– 6.4
C00001612-37.98	– 6.6	C02007680-40.60	– 6.4
C00001732-38.46	– 7.4	C02007682-40.07	– 5.9
C00049153-34.88	– 5.7	C02522669-39.12	– 8
C00083315-40.39	– 6.5	C02583773-40.82	– 6.4
C00105196-35.68	– 5.9	C03830195-38.08	– 6.2
C00139370-39.46	– 6.2	C03830716-45.01	– 8.4
C00404450-38.34	– 5.8	C03830891-36.56	– 5.1
C00404451-38.21	– 5.8	C03831014-38.17	– 6.7
C00537752-43.31	– 6.6	C03875336-39.88	– 7.3
C00601281-42.46	– 8.1	C04213409-38.64	– 7.7
**C00601317-37.80**	**– 8.8**	**C04214975-41.34**	**– 8.6**
C00608179-37.64	– 8.1	C04214989-39.73	– 8.1
C00895032-33.50	– 4.9	**C04215004-38.87**	**– 9.3**
C00895034-33.43	– 4.2	C04215196-42.02	– 8.1
C00968306-38.11	– 6.3	C04215955-38.54	– 7.1
C00968311-37.53	– 7.1	C04215962-37.88	– 6.8
C01530763-39.83	– 6.1	C04228231-45.74	– 6.8
C01530767-39.44	– 6.4	C05458868-37.76	– 7.9
C01532129-47.08	– 6.4	C05458875-40.26	– 7.6
C01532130-57.63	– 6.1	C05458878-42.73	– 8.1
C01532131-48.83	– 6.4	C05458886-39.86	– 8.2
C01532132-51.30	– 6.2	C05458893-37.83	– 7.3
C01532522-43.70	– 4.8	C05458899-42.51	– 7.7
C01532525-45.84	– 4.4	C05650525-41.60	– 7.9
C01532526-36.79	– 5.1	C05843973-38.23	– 7.1
C01532529-33.17	– 3.5	C06482440-38.48	– 6.7
C01532530-40.74	– 4.6	C08221225-33.47	– 7.9
C01532556-39.26	– 4.9	C12484881-45.91	– 4.9
C01532614-39.14	– 5.4	C12484909-39.08	– 5.9
C01532673-49.49	– 4.2	C12495103-40.98	– 5.7
C01532734-35.55	– 4.3	C12500917-42.62	– 7
C01587665-38.97	– 6.2	**C12500925-38.35**	**– 8.4**
C01608965-40.22	– 6.5	C12500934-43.56	– 8.2
C01664586-40.34	– 7.9	C12500936-43.57	– 8.1
C01664587-42.32	– 7.9	C13513942-35.19	– 8
C01841010-37.79	– 5.4	C13513943-35.91	– 7.8
C01849735-37.93	– 7.3	C33938186-33.64	– 7.9
C01850015-38.24	– 6.4	C40017481-38.50	– 7.6
C01850017-40.11	– 7.4	C00000441-37.59	– 5.7
C01850106-41.77	– 7.1	C00000859-37.64	– 5.7
C01087483-37.50	– 6.4	C00001612-37.98	– 6.5
C03830195	– 6.2	C00601317-37.80	– 8.9
C04215962-37.88	– 6.9	C05458868-37.76	– 7.9

Lowest binding energy ZINC ligand selected for study marked in bold

**Table 3:** Docking interactions between four complexes of CathD- C1, CathD-C2, CathD-C3 and CathD-C4

Sr. No	Complex	Interactions	Distance in Å
1	CathD-C1	C:ABY242:H20—A:ASP33:OD1 C:ABY242:N20—B:ASP126:OD2 C:ABY242:H153—B:ASP126:OD2 B:TYR100—C:ABY242 C:ABY242—A:VAL31 C:ABY242—B:ILE124	2.01 3.98 2.09 4.69 5.23 5.34
2	CathD-C2	C:AAI242:H9—A:ALA13:O A:GLN14:HA—C:AAI242:O41 C:AAI242:H72—B:GLY128:O C:AAI242:H73—B:GLY128:O C:AAI242:H383—B:THR127:O C:AAI242:H443—B:THR127:O A:TYR15:HB2—C:AAI242	1.86 2.26 2.65 3.08 2.90 2.57 2.68
3	CathD-C3	C:AAW242:H292—A:TYR78:O C:AAW242:H262—A:TYR78:O	2.60 2.33
4	CathD-C4	C:ABO242:H252—B:ASP218:OD1 C:ABO242:H253—B:THR127:O C:ABO242:H3—B:GLY128:O C:ABO242:H12—A:ALA13:O C:ABO242:H13—A:ALA13:O C:ABO242:H222—B:GLY128:O C:ABO242:H222—B:SER130:OG C:ABO242:H28—A:ALA13:O	1.69 1.65 2.99 2.92 2.73 2.10 2.58 2.17

### Docking using PyRx and AutoDock

**Fig. 2:**
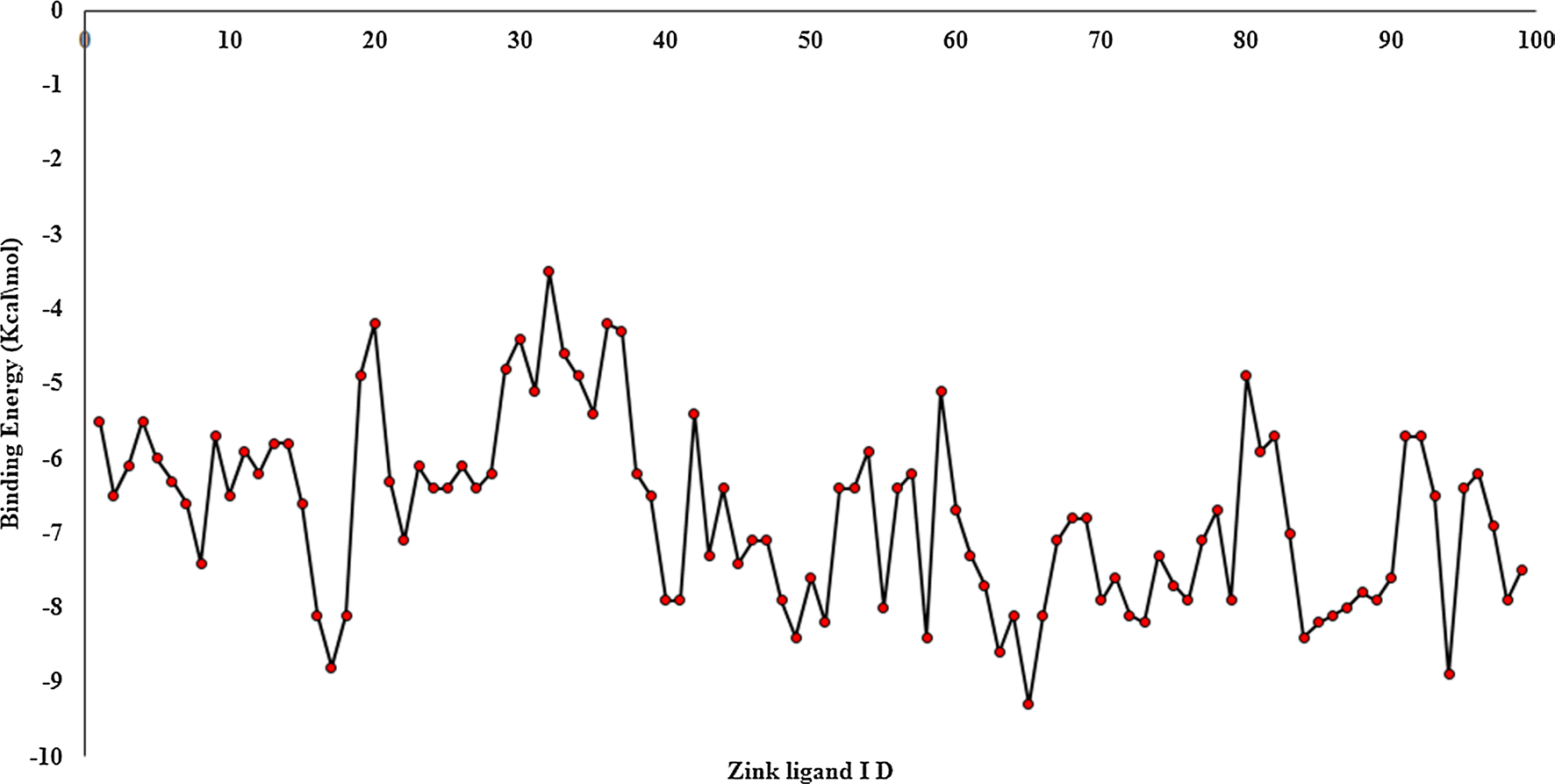
Binding energy of ligand with whole CathD given by AutoDock

**Fig. 3:**
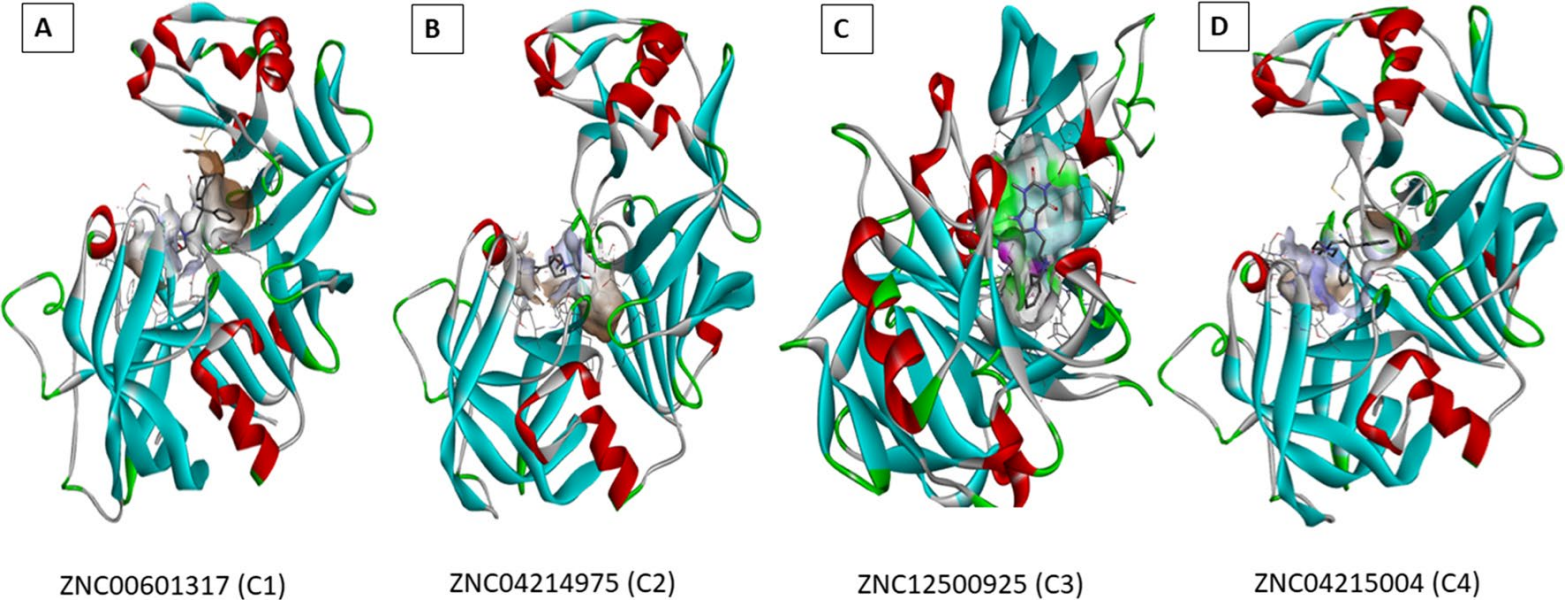
The docked complexes as of CathD-C1, CathD-C2, CathD-C3 and CathD-C4

The preferred binding site obtained using blind docking at the interface of two subunits of CathD has been further confirmed by performing local docking using PyRx. All the 100 compounds occupy the same binding pocket, i.e. interface of the heterodimeric subunits and exhibit conserved binding site interactions with the residues mentioned above. Amongst all the interacting residues, we observed the residues Asp33, Asp126 showing maximum participation in the stable non-bonded interactions. Thus, we propose that being an aspartate protease, Asp33, Asp126 are the most significant residues of CathD facilitating stable complex of the CathD with the selected top 100 compounds. Table 2 represents the binding free energy values in kcal/mol of individual compounds given by the PyRx docking. The binding mode and energy of top 100 compounds has been shown in Figs. 1 and 2. The binding energy of docked complexes of C1-CathD is − 8.8 kcal/mol (Fig. 3A), C2-CathD is − 8.6 kcal/mol (Fig. 3B), C3-CathD is − 9.4 kcal/mol (Fig. 3C) and C4-CathD is − 8.4 kcal/mol (Fig. 3D) after docking. The structural stability of the top four molecules representing least binding energy and maximum number of non-bonded interactions has been analysed using MD simulations. The detailed intermolecular interactions for these best four compounds before MD are shown in Table 3 and detailed intermolecular interactions for these best four compounds after MD are shown in Table 4.

**Table 4:** Interactions after MD simulation between four complexes CathD- C1, CathD-C2, CathD-C3 and CathD-C4

Sr. No	Complex	Interactions	Distance in Å
1	CathD-C1	C:ABY242:H_2_0—A:ASP33:OD1 B:GLY128:HA3—C:ABY242:O19 C:ABY242:H173—A:ASP33:OD1 C:ABY242:H143—A:ASP33:OD2 C:ABY242:H152—B:ASP126:OD1	1.79 2.54 2.90 2.98 2.50
2	CathD-C2	C:AAI242:H9—A:ALA13:O A:GLN14:HA—C:AAI242:O41 C:AAI242:H43—B:GLY128:O	1.85 2.72 2.29
3	CathD-C3	C:AAW242:H272—B:THR129:OG1 C:AAW242:H273—B:ASP126:OD2	2.64 2.26
4	CathD-C4	B:SER130:H—C:ABO242:O8 B:SER130:HG—C:ABO242:O8 C:ABO242:H253—B:THR129:O B:THR129:HA—C:ABO242:O8 C:ABO242:H12—B:THR127:O C:ABO242:H13—B:THR127:O C:ABO242:H28—B:THR127:O C:ABO242:H143—B:GLY128:O C:ABO242—A:VAL31	1.77 2.81 1.76 1.87 2.81 2.67 2.35 2.85 3.03

The receptor structure of whole CathD was prepared in AutoDock wizard. The Kollman united atom charges were assigned to the receptor atom. The charges and protonation states of active site and interacting residues were properly assigned as per the previous results of docking studies (Huo 2002; Irwin et al. 2005; Gacko et al. 2007). The 100 optimised ligand hits were redocked using PyRx software with whole CathD receptor. The generated docked conformations were analysed for binding energy, intermolecular energy and internal energy of each conformation as observed and clustered within 2.0 Å (Fig. 1). The top ranking four ligands (ZINC00601317, ZINC04214975, ZINCC12500925, ZINCC04215004) and receptor complexes were analysed for hydrogen bonding, hydrophobic and hydrophilic interactions.

The ligands ZINC00601317, ZINC04214975, ZINCC12500925 and ZINCC04215004 were redocked with whole CathD with flexible residues Asp33, Tyr78, Asp231, Ser36, Ser80, Thr234, Thr205, Gly233, Gly81 using AutoDock 4.2 (Morris et al. 2009). Grid size was redefined which is centred on selected flexible residues. The grid size was set to 40 X52 X 45 points with a grid spacing of 0.375 Å centred on the selected flexible residue present in active site of enzyme. The grid box includes the entire binding site of the enzyme and provides enough space for the ligand translational and rotational walk. After 270,000 LGA operations, the generated conformations were selected on the basis of lowest binding energy and clustered RMSD less than 1 Å.

### Molecular dynamic (MD) simulation

**Fig. 4:**
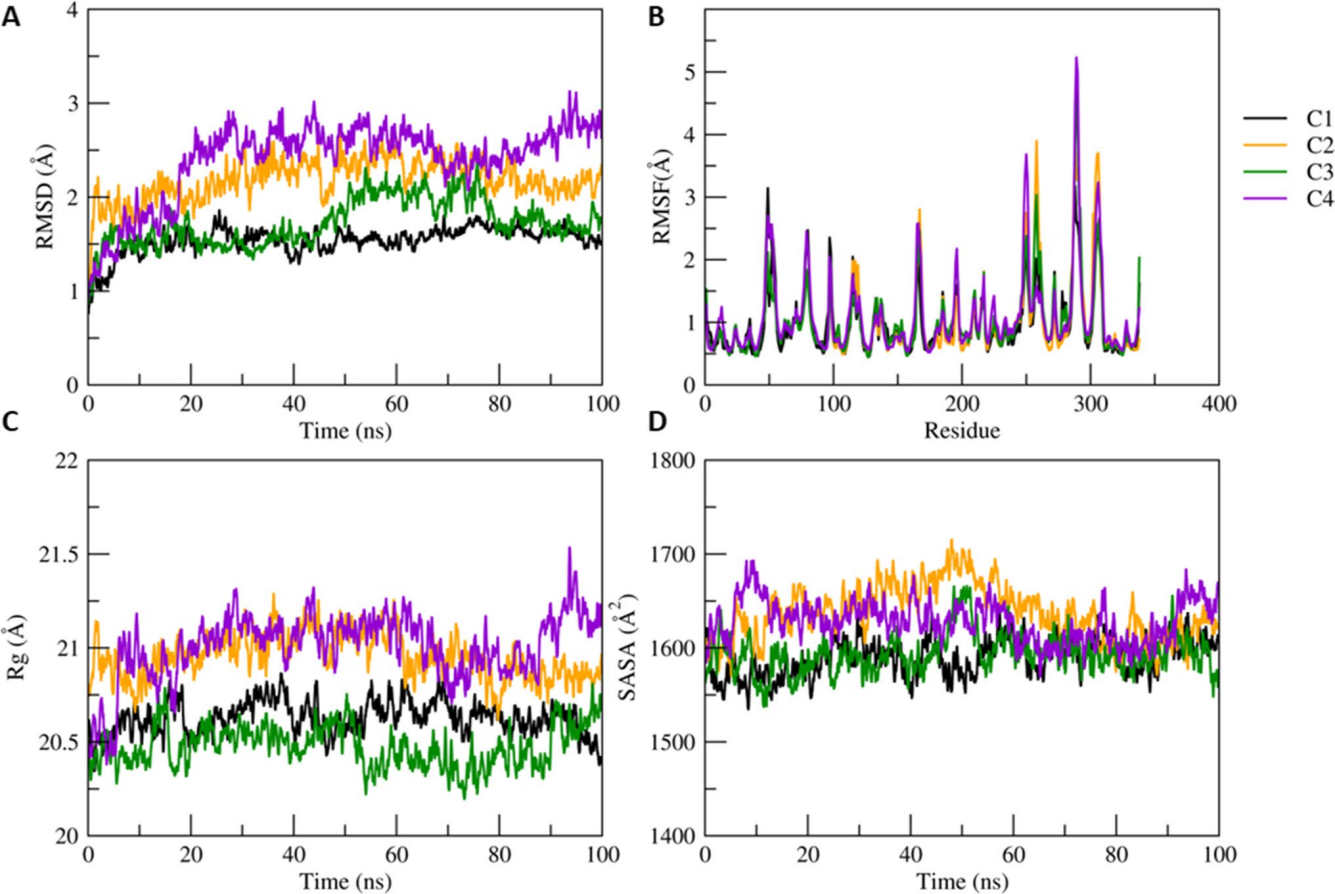
**A** RMSD, **B** RMSF, **C** Rg, **D** SASA of all four docked complexes CathD-C1, CathD-C2, CathD-C3 and CathD-C4

**Fig. 5:**
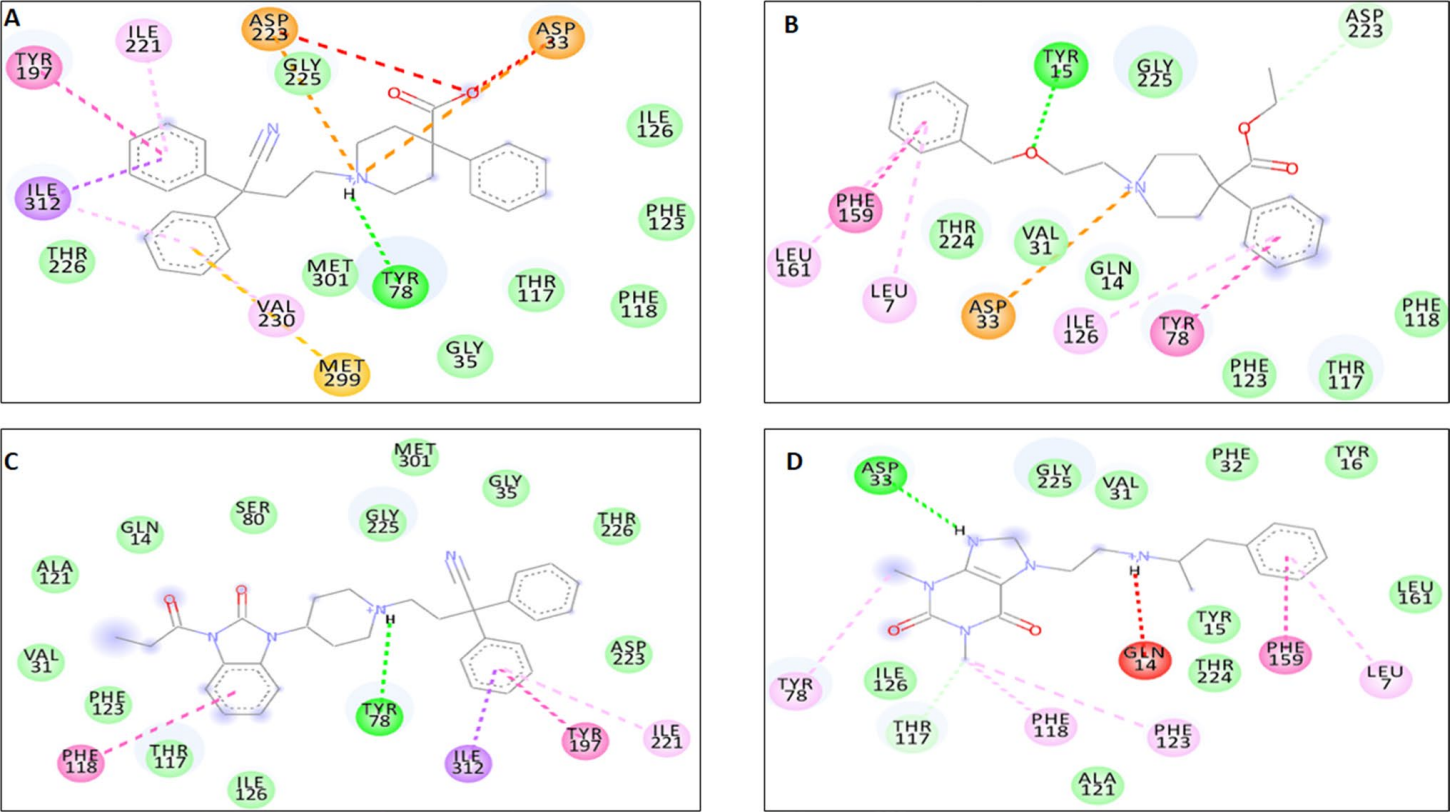
**A** Interacting residues of CathD with ZINC database molecules ZINC00601317 **(C1)**; **B** Interacting residues of CathD with ZINC database molecules ZINCC04214975 **(C2); C** Interacting residues of CathD with ZINC database molecule ZINCC04215004 **(C3); D** Interacting residues of CathD with ZINC database molecules ZINCC12500925 **(C4)**

Structural stability of the docked complexes CathD-C1, CathD-C2, CathD-C3 and CathD-C4 has been monitored during 100 ns simulations. The time-dependent evolution of backbone RMSD value for all the simulated complexes is less than 3 Å which suggests that the equilibrium state has been achieved after 40 ns (Fig. 4A). The stable RMSD values observed are in this order; CathD-C1 < CathD-C3 < CathD-C2 < CathD-C4. The residual fluctuations plotted for C-alpha atom show maximum fluctuations of ~ 5.2 Å for complex CathD-C4. The binding pocket residues Asp33, Asp126, Gly35, Val31, Thr34, Gly128, Ile124, Ala13 Ser130, Thr127, Gln14 show lesser RMS fluctuations (< 1 Å) as they participate in the formation of stable non-bonded interactions. The maximum fluctuations are expressed by the flexible loops and/or turns exhibited by the heterodimeric subunits. The binding pocket is located at the inter-dimer interface and all the compounds (C1, C2, C3 and C4) favour the binding pocket region of CathD and fits well into the cavity throughout the MD simulation. The complexes CathD-C1 and CathD-C2 show compact folding as revealed by steady decrease in the Rg value. Partial unfolding has been observed in complexes CathD-C3, CathD-C4, structural analysis over the trajectory shows that the inter-dimer interface undergoes significant conformational changes that would favour stable complex formation either by folding or unfolding the heterodimeric interface. The C4 goes deeper into the CathD heterodimeric inter-dimer interface by undergoing the structural unfolding mainly at the binding pocket region (dimer interface). The N-terminal short helix formed by the residues (Ile19 to Ala23 of chain B) loses its helicity causing the increase in its conformational flexibility resulting in the enhanced interactions of C3 to the inner core of the CathD interface (Fig. 3C). The larger sized molecules C1 and C3 express extended conformation across the binding to the interface of the two subunits. Further, exposure of hydrophobic residues to the solvent has been estimated by calculating the solvent-accessible surface area (Fig. 4D). The SASA values for all the complexes were converged after the 60 ns to ~ 1600 Å^2^ (or 160 nm^2^) suggesting the compact folding upon stable complex formation. However, SASA value for complex CathD-C4 increases to 1650 Å^2^ after suggesting exposure of the binding pocket residues. We propose that this conformational change at the binding pocket region is necessary to accommodate large sized molecule C4 in the CathD inter-dimer interface. It has been observed that binding pocket interactions for complex CathD-C3 show no significant increase or decrease in the non-bonded contacts in particular hydrophobic, electrostatic and H-bonding interactions during the simulation. Hence, C3 represents weaker binding affinity to the CathD when compared to other simulated complexes. Whilst in complexes CathD-C2 and CathD-C4, we observed dominating hydrophobic and H-bonding interactions during the simulation period. In all the complexes, we observed hydrophobic residues namely Gly35, Val31, Thr34, Gly128, Ile124 and Ala13 exhibit conserved binding pocket interactions. Moreover, negatively charged residues Asp33 and Asp126, also maintain the conserved non-bonded interactions throughout the MD simulation. Thus, it has been noticed that residues located at the inter-dimer interface of CathD significantly contributes to stable dimerization as well as complex formation with the screened compounds (Fig. 5).

### MM/GBSA binding free energy calculation

We used MM/GBSA approach to calculate binding free energy between CathD and selected compounds, e.g. C1, C2, C3, C4. The order of binding affinity has been estimated as CathD-C2 (− 38.90 kcal/mol)—< CathD-C4 (− 30.20 kcal/mol) < CathD-C1 (− 25.45 kcal/mol) < CathD-C3 (− 25.09 kcal/mol). The compound C2 fits well to the inter-dimer interface and gets deeply buried during the MD simulation causing the increase in the non-bonded interactions. Table 5 represents the various energy components of the binding free energy. The residue decomposition energy also plotted for all the four complexes, it reveals that the residues from the binding cavity and heterodimeric subunit interface residues help to maintain architecture of the binding pocket showing maximum contribution in the calculated binding free energy. In particular, residues Asp33, Asp231, Ser36, Ser80, Thr234, Thr205, Gly233 and Gly81 show conserved binding pocket interactions in all the studied complexes. Hence, we propose that these are the key significant residues of the CathD promotes stable complex formation by undergoing the local secondary structural changes at the heterodimeric interface region.

**Table 5:** Binding energy component, total binding energy of four complex of CathD with ZINC00601317, ZINC04214975, ZINCC12500925 and ZINCC04215004

Complexes	ΔEVDW	ΔEEL	ΔEGB	ΔESURF	ΔGGAS	ΔGSOLV	ΔTOTAL
CathD-C1	– 35.06 ± 8.01	– 42.16 ± 1.68	56.59 ± 2.19	– 4.82 ± 1.13	– 77.21 ± 8.18	51.77 ± 2.46	– 25.45 ± 8.55
CathD-C2	– 33.99 ± 3.81	– 36.40 ± 3.17	35.85 ± 3.63	– 4.37 ± 0.65	– 70.38 ± 4.96	31.48 ± 3.69	– 38.90 ± 6.18
CathD-C3	– 34.74 ± 7.94	– 144.47 ± 27.16	158.62 ± 30.83	– 4.50 ± 1.07	– 179.21 ± 28.30	154.12 ± 30.85	– 25.09 ± 9.53
CathD-C4	– 34.89 ± 5.37	– 226.46 ± 27.49	235.78 ± 25.27	– 4.64 ± 0.71	– 261.34 ± 29.88	231.15 ± 24.98	– 30.20 ± 5.64

MM-PBSA of four complexes in Kcal/Mol

## Conclusion

Thus, in the present study, molecular modelling approaches have been successfully employed to identify the key binding site residues of CathD. The residues Asp33, Asp231, Ser36, Ser80, Thr234, Thr205, Gly233 and Gly81 are the most significant and favour the stable complex formation. The inter-dimer interface between the two heterodimeric subunits show proper folding during the MD simulation forming the deep and extended cavity that accommodates larger molecules easily. Binding site identified in the present study would pave the way to explore the mechanism of CathD-dependent degradation of amyloid beta. Thus, this study would be helpful to identify active site pocket of CathD, which could be beneficial to develop the novel therapeutic strategies for the Alzheimer’s disease caused by amyloid beta peptides.

## Data Availability

All data underlying the results are available as a part of article and no additional source data is required.
